# Comparative Functional Analysis Reveals Conserved Roles of Aquaporins Under Osmotic Dehydration in *Steinernema carpocapsae* Strains

**DOI:** 10.3390/biology15010078

**Published:** 2025-12-31

**Authors:** Yongqi Chen, Qiuyue Huang, Xun Yan

**Affiliations:** 1 College of Agriculture and Biology, Zhongkai University of Agriculture and Engineering, Guangzhou 510225, China; 2 Key Laboratory of Green Prevention and Control on Fruits and Vegetables in South China, Ministry of Agriculture and Rural Affairs, Guangzhou 510225, China; 3 Innovative Institute for Plant Health, Zhongkai University of Agriculture and Engineering, Guangzhou 510225, China

**Keywords:** aquaglyceroporin, aquaporin, osmotic dehydration, phylogenetic analysis, *Steinernema carpocapsae*, expression pattern

## Abstract

Entomopathogenic nematodes (EPNs), including *Steinernema* and *Heterorhabditis*, are obligate insect parasites widely used in biological pest control. However, their efficacy is often limited by susceptibility to environmental stresses like desiccation. Aquaporins (AQPs), channel proteins facilitating water and solute movement across membranes, are hypothesized to play a key role in the osmotic stress response of EPNs. This study identified and cloned three AQP genes (L596_g7661, L596_g18121, and XLOC_007750) from four strains of *Steinernema carpocapsae*. Bioinformatic analysis confirmed that these AQPs belong to the aquaglyceroporin subfamily and share high sequence homology across strains. Functional characterization in *Xenopus* oocytes demonstrated that AQP L596_g7661 facilitates glycerol transport. Expression patterns under osmotic dehydration revealed significant upregulation of L596_g7661 and XLOC_007750 in all strains, while L596_g18121 expression remained unchanged. These findings indicate that specific AQPs are involved in the molecular response of *S. carpocapsae* to osmotic stress, providing crucial insights for breeding resilient EPN strains and enhancing their field application.

## Simple Summary

This study investigated the role of aquaporins (AQPs) in the osmotic stress response of the entomopathogenic nematode *Steinernema carpocapsae*. Three AQP genes (L596_g7661, L596_g18121, and XLOC_007750) were cloned from four different *S. carpocapsae* strains. Bioinformatic analysis confirmed they belong to the aquaglyceroporin subfamily. Functional assay in *Xenopus* oocytes demonstrated that the AQP L596_g7661 facilitates glycerol transport. Under osmotic dehydration, the expressions of L596_g7661 and XLOC_007750 were significantly upregulated across all strains, while L596_g18121 expression remained unchanged. The findings reveal that specific aquaglyceroporins are involved in the molecular adaptation of *S. carpocapsae* to osmotic stress. This research provides a theoretical basis for identifying key stress-tolerance genes and contributes to the future breeding of resilient nematode strains for more reliable biological pest control.

## 1. Introduction

Entomopathogenic nematodes (EPNs), including *Steinernema* and *Heterorhabditis*, are obligate parasites of insects. EPNs can effectively infect and kill a wide range of insects and are consequently employed in the biological control of insect pests, particularly those that are soil-dwelling and boring [1,2,3]. They are recognized for their safety toward the environment, plants, and non-target organisms [4]. The production and marketing of EPNs have reached a commercial scale [5]. However, in practical field applications, the viability of EPNs is highly susceptible to adverse environmental factors such as desiccation, extreme temperatures, hypoxia and UV radiation, leading to a decline in efficacy [3]. Under environmental stresses like drought and high osmotic pressure, nematodes undergo dehydration, which directly reduces their control efficacy and ultimately limits their large-scale application. Studies have found that suitable osmotic dehydration could induce partial anhydrobiosis in some EPN strains and significantly enhance their cold and heat tolerance [6,7,8]. Dehydration triggers the synthesis of numerous stress-related proteins and initiates extremely complex molecular reactions in EPN [7,8,9,10,11,12]. However, the complete molecular mechanism underlying the dehydration response remains to be fully elucidated.

Aquaporins (AQPs) are channel-forming proteins that specifically facilitate the permeation of water and certain small solutes, such as glycerol and urea [13]. They play important roles in the cell membrane, including facilitating transmembrane water transport, osmoregulation, nutrient absorption, and the excretion of toxic metabolites [14,15]. For instance, AQPs in rice (*Oryza sativa*) have critical functions in cellular osmoregulation and ion homeostasis maintenance [16]. In nematodes, AQPs in *Caenorhabditis elegans* play a key role in maintaining salt/water balance during hyper-osmotic stress [17]. The AQP gene family has been predicted from the genome and transcriptome data of the EPN *Steinernema carpocapsae* in response to desiccation and ultraviolet radiation stress [18]. Nevertheless, it remains unclear whether the AQPs in *S. carpocapsae* share similar functions to those in *C. elegans* or other organisms, or whether they are involved in the adaptation of EPN to dehydrating environments.

This study identified and cloned three AQP genes from different *S. carpocapsae* strains. It is the first report to conduct a side-by-side comparison of AQP genes across different strains within the same nematode species, revealing conserved sequence features amid subtle variations. Furthermore, this work constitutes the first functional validation of an AQP gene in any EPN, demonstrating the glycerol transport activity of AQP L596_g7661 through heterologous expression in *Xenopus* oocytes. By integrating comparative genomics with molecular functional analysis and by linking specific AQP expression patterns to osmotic stress response, this research provides foundational insights into the molecular mechanisms of environmental adaptation in this biologically important organism.

## 2. Materials and Methods

### 2.1. Nematodes

The *Steinernema carpocapsae* strain All, 92-2, and G-R3a-2 were maintained in the laboratory. *S. carpocapsae* Nema-ky was purchased from Keyun Biology (Jiyuan, China). Infective juveniles (IJs) of each strain were produced by in vivo culture in the greater wax moth (*Galleria mellonella*) following the method of Kaya and Stock [19]. Harvested IJs were stored at 10 °C and used within two weeks. The viability of IJs was confirmed on the day of experimentation, with a requirement of <5% mortality. Different batches of nematodes were used for repeated bioassays, with each assay conducted at least twice.

### 2.2. Osmotic Treatment

IJs were treated with an osmotic solution and incubated at 15 °C for 72 h in a rotary shaker at 100 rpm. The osmotic solution is a mixture of glycerol (6.4 M) and fortified artificial seawater (1692.00 mM NaCl, 9.00 mM KCl, 9.27 mM CaCl_2_, 22.94 mM MgCl_2_·6H_2_O, 25.50 mM MgSO_4_·7H_2_O, 2.14 mM NaHCO_3_) at a proportion of 5:1 (*v*/*v*) [20]. The survival of IJs after recovery in distilled water for 24 h was assessed after 24, 48 and 72 h of osmotic treatment as described previously [7]. The infectivity of the recovered IJ against last instar larvae of *G. mellonella* was evaluated as mentioned [21]. For molecular analyses, osmotically treated IJ samples were collected at 0 h, 3 h, 12 h, 18 h and 24 h for RNA extraction, which were used for gene cloning and quantitative RT-PCR.

### 2.3. Gene Cloning

Total RNA was extracted from the osmotically treated and untreated IJs and reverse transcribed into cDNA. Primers were designed based on the predicted AQP sequences (L596_g7661, L596_g18121, and XLOC_007750; GenBank Accessions Nos. PQ643875-PQ643877) and used to clone the AQP genes from the different *S. carpocapsae* strains (Table 1). The primer concentration used was 10 μM. Target genes were amplified using 2×TransTaq^®^ High Fidelity (HiFi) PCR SuperMix (TransGen Biotech, Beijing, China). The 50 μL reaction system consisted of 25 μL of 2×TransTaq^®^ High Fidelity (HiFi) PCR SuperMix, 1 μL of each primer (10 μM), 2 μL of cDNA, and 21 μL of nuclease-free water. The PCR amplification protocol was as follows: initial denaturation at 94 °C for 5 min; 35 cycles of denaturation at 94 °C for 30 s, annealing at 50–60 °C for 30 s (according to the Tm of the specific primer pair), and extension at 72 °C for 1 min; followed by a final extension at 72 °C for 7 min. The cloned AQP cDNAs were sequenced and submitted to GenBank.

### 2.4. Bioinformatic and Phylogenetic Analysis

The translated protein sequences of the cloned AQP genes were analyzed for physicochemical properties using the ExPASy server (https://www.expasy.org/resources/protparam (accessed on 5 March 2025)). Transmembrane domains were predicted using TMHMM 2.0 (http://www.cbs.dtu.dk/services/TMHMM/ (accessed on 5 March 2025)), and conserved domains were identified using the NCBI Conserved Domain Database (https://www.ncbi.nlm.nih.gov/cdd/ (accessed on 5 March 2025)). A phylogenetic tree of the *S. carpocapsae* AQPs was constructed alongside *C. elegans* aquaporins AQP1 to AQP8 using the Neighbor-Joining method.

### 2.5. Xenopus Oocyte Transport Studies

The aquaporin L596_g7661 and green fluorescent protein (*gfp*) gene fragments were cloned using primers listed in Table 1. Each fragment’s end was equipped with a 15-bp homologous arm. The PCR products were ligated into the pGEMHE vector after double digestion with BamHI and XbaI. The ligated plasmids were screened, verified and extracted. Capped cRNAs were synthesized in vitro from the linearized plasmids and microinjected into defolliculated *Xenopus* oocytes. The expression of the AQP gene and GFP in oocytes was confirmed by the presence of green fluorescent on the cell membranes using confocal microscopy (Zeiss LSM900 Airyscan 2, Oberkochen, Germany). The osmotic water permeability (*P_f_*) was determined by measuring the rate of oocyte swelling upon transfer to a 1:3 diluted ND96 buffer. Glycerol permeability was assessed based on the initial swelling rates after incubating the oocytes in an isosmotic ND96 solution where NaCl was replaced by glycerol [13,22].

### 2.6. Gene Expression Analysis

The expression levels of the AQP genes L596_g7661, L596_g18121, and XLOC_007750 were quantified in four *S. carpocapsae* strains following osmotic dehydration for different duration (0 h, 3 h, 6 h, 12 h, 18 h, 24 h) using RT-qPCR. Gene-specific qPCR primers were designed based on the cloned AQP sequences (Table 1). All amplification products were sequenced to confirm target specificity. For each RNA sample (extracted from osmotically treated and control IJs), 400 ng was reverse-transcribed into cDNA using TransScript^®^ One-Step gDNA Removal and cDNA Synthesis SuperMix (TransGen Biotech, Beijing, China). The synthesized cDNA was then subjected to qPCR analysis using PerfectStart^®^ Green qPCR Super (TransGen Biotech, Beijing, China). The 20 μL reaction mixture contained 10 μL of PerfectStart^®^ Green qPCR SuperMix, 0.4 μL of each primer (10 μM), 2 μL of cDNA, and 7.6 μL of nuclease-free water. The PCR cycling conditions were: 94 °C for 30 s, followed by 40 cycles of 94 °C for 5 s and 60 °C for 30 s. Actin (amplified with primers listed in Table 1) served as the reference gene. The fold difference in AQP gene expression between osmotically treated and control nematodes was analyzed using comparative quantification [8].

### 2.7. Statistical Analysis

Nematode survival rates were calculated and corrected. Relative gene expression levels were determined using the 2^−ΔΔCt^ method [8]. Means and standard errors were computed for both survival rates and gene expression levels. Statistical significance was assessed using a Paired-sample *t*-test and One-way ANOVA in SPSS software 27.0. In all analyses, differences with *p* < 0.05 were considered statistically significant.

## 3. Results

### 3.1. AQP Genes in Different S. carpocapsae Strains

Full-length cDNA sequences of three AQP genes, L596_g7661, L596_g18121 and XLOC_007750 were cloned from four *S. carpocapsae* strains: Nema-ky, All, 92-2, and G-R3a-2. The respective cDNA lengths were 864 bp, 906 bp and 930 bp. The cloned sequences (GenBank Accessions Nos. PV955126-PV955137) exhibited high homology to the corresponding coding sequences in GenBank. The same AQP gene had identical cDNA lengths across the different strains. Sequence identity rates for the same AQP gene across strains were 99.65% for L596_g7661, 99.70% for L596_g18121, and 99.78% for XLOC_007750.

### 3.2. Amino Acid Sequences of Aquaporins

**Figure 1:**
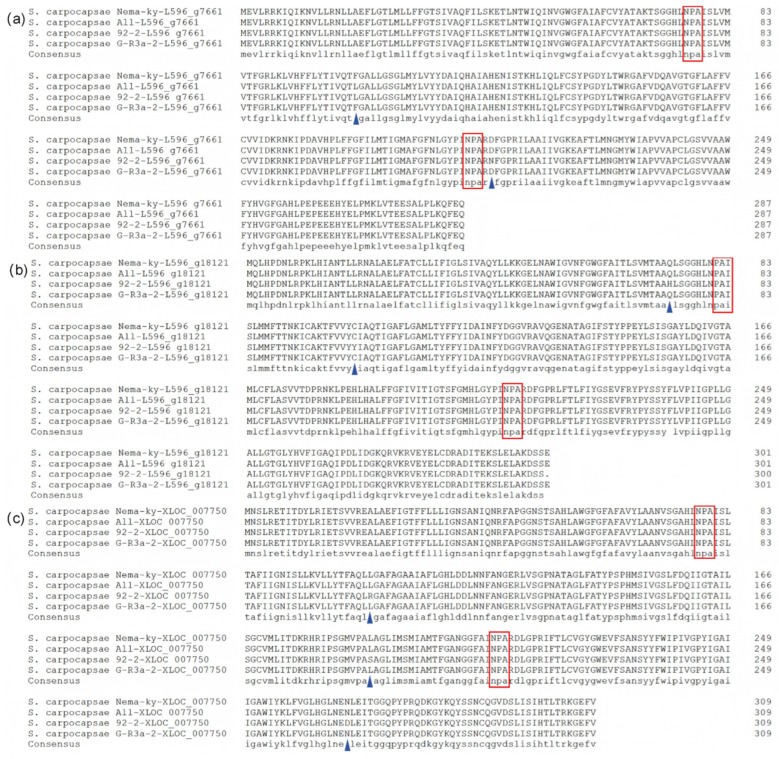
Amino acid sequence alignment of aquaporins from different *Steinernema carpocapsae* strains. (**a**), L596_g7661; (**b**), L596_g18121; (**c**), XLOC_007750. Boxes indicate the conserved NPA motifs. Triangles denote positions of amino acid variations.

The deduced amino acid sequences of L596_g7661, L596_g18121, and XLOC_007750 consisted of 287, 301 and 309 residues, respectively. Although variations existed among strains, all sequences contained two highly conserved asparagine–proline–alanine (NPA) motifs characteristic of AQPs (Figure 1). The amino acid sequence identity for the same AQP across different strains were 99.74% for L596_g7661, 99.67% for L596_g18121, and 99.76% for XLOC_007750.

### 3.3. Physicochemical Properties of Aquaporins

Analysis of the physicochemical properties is summarized in Table 2. The molecular weights of the AQPs from the four strains ranged from 32.05 kDa to 33.21 kDa. Their instability indices ranged from 21.58 to 27.41, classifying them as stable proteins. The isoelectric points (pI) varied between 6.37 and 7.73, indicating they are weakly acidic. The average hydrophilicity indices ranged from 0.403 to 0.578, confirming their hydrophobic nature.

### 3.4. Transmembrane and Conserved Domains of AQPs

Conserved domain analysis using the NCBI CDD revealed that all three AQPs from the four *S. carpocapsae* strains possess the typical structural characteristics of the Major Intrinsic Protein (MIP) family (Figure S1). Transmembrane domain prediction indicated that these genes are integral membrane proteins, each containing six transmembrane domains (Figure S2), consistent with the canonical aquaporin structure.

### 3.5. Phylogenetic Analysis of AQPs in S. carpocapsae

**Figure 2:**
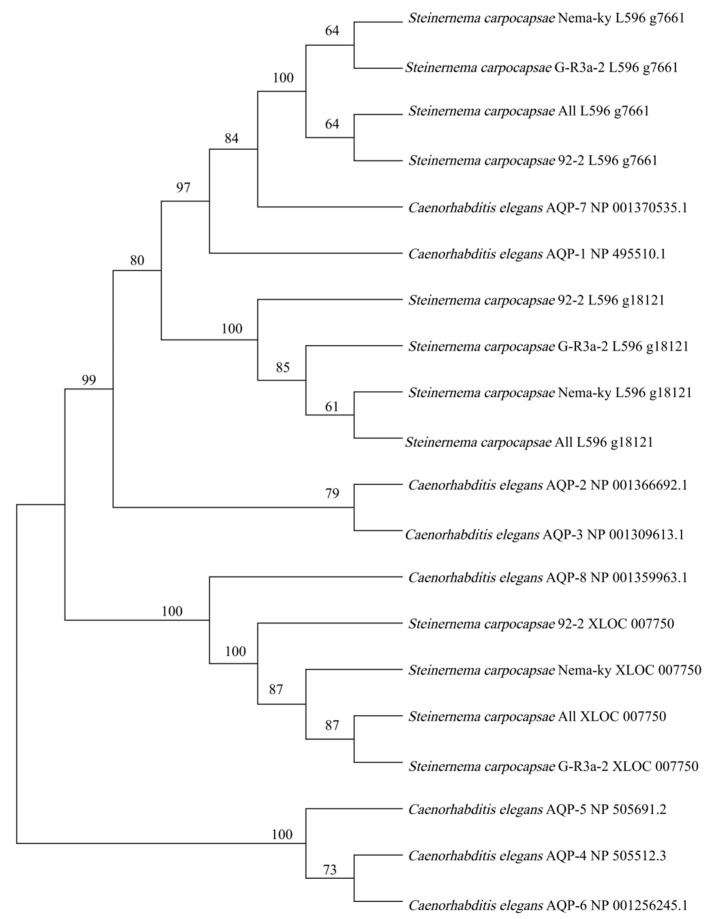
Phylogenetic tree of aquaporins from *Steinernema carpocapsae*. The tree was constructed using the Neighbor-Joining method. Bootstrap values from 1000 replicates are shown at the nodes.

A phylogenetic tree was constructed using the amino acid sequences of the three AQPs from the four *S. carpocapsae* strains and *C. elegans* AQP1 to AQP8 (GenBank Accessions Numbers provided in Figure 2). L596_g7661 and L596_g18121 from all *S. carpocapsae* strains clustered closely with *C. elegans* AQP1 and AQP7. XLOC_007750 formed a cluster with *C. elegans* AQP8. All these AQPs belong to the aquaglyceroporin subfamily, which transports both water and small solutes like glycerol.

### 3.6. Survival of Different S. carpocapsae Strains Under Osmotic Dehydration

**Figure 3:**
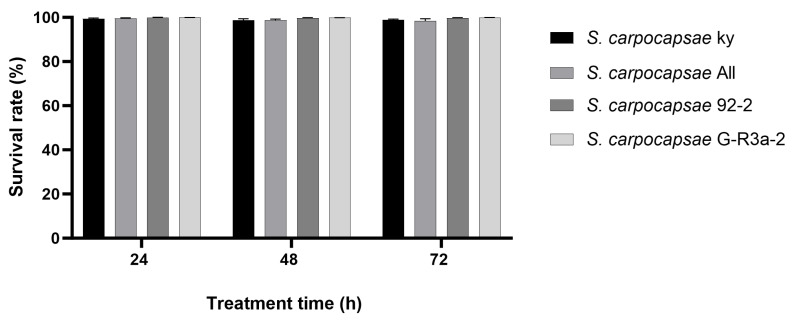
Survival rates of different *Steinernema carpocapsae* strains after osmotic treatment. No significant differences in survival rates were observed among strains and treatment durations (One-way ANOVA, *n* = 6, *p* > 0.05).

The corrected survival rates of the four strains after 24 h, 48 h, and 72 h of osmotic treatment are shown in Figure 3. All strains exhibited survival rates higher than 97%. No significant differences in survival were found between the osmotic treatment groups and the control at any treatment time point (*t* ≤ 1.150, *p* ≥ 0.302). Survival rates did not show a significant difference among strains and treatment durations (*F* = 1.555, df = 11, 60, *p* = 0.136). The infectivity of the osmotically treated IJs after recovery did not differ from that of IJs stored in water. All IJs caused 100% mortality to the last instar larvae of *G. mellonella* after 48 h of infection.

### 3.7. Glycerol Permeability of AQP L596_g7661

**Figure 4:**
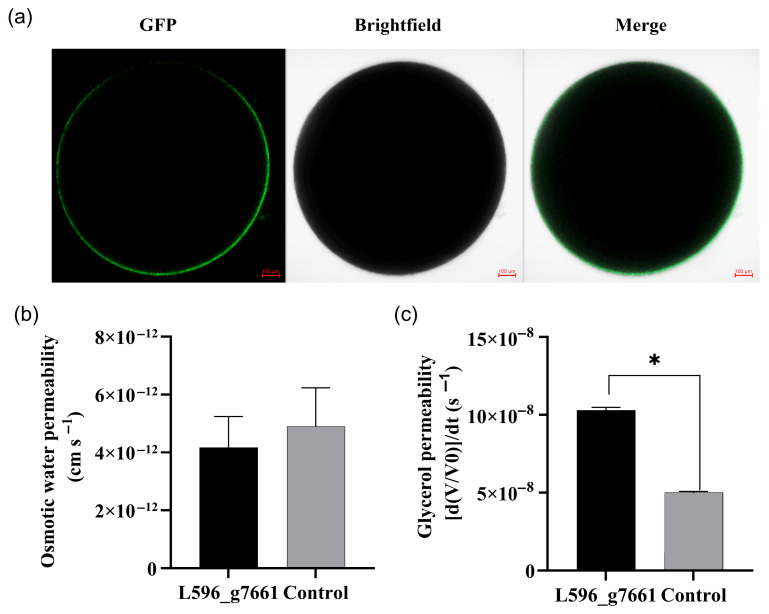
Functional characterization of aquaporin L596_g7661 in *Xenopus* oocytes. (**a**), Representative images of an oocyte expressing the AQP L596_g7661-GFP fusion protein. From left to right: GFP (fluorescence signal of the fusion protein), Brightfield (DIC image), and Merge (overlay of both channels). The scale bar represents 100 µm. (**b**), Osmotic water permeability (*Pf*) of oocytes expressing AQP L596_g7661 compared to water-injected controls. (**c**), Glycerol permeability of oocytes expressing AQP L596_g7661. Asterisk (*) above the bars indicates a significant difference between the oocytes expressing AQP L596_g7661 and the control (Paired-sample *t*-test, *n* = 7, *p* < 0.05).

Co-expression of AQP L596_g7661 and GFP in *Xenopus* oocytes resulted in green fluorescent on cell membrane (Figure 4a). In oocyte swelling assays using a 1:3 diluted ND96 buffer, the water permeability of oocytes expressing AQP L596_g7661 was not significantly different from the control (*t* = 0.395, *p* = 0.706) (Figure 4b). However, after incubation in a glycerol solution for 20 s, the initial swelling rate of oocytes expressing AQP L596_g7661 was 0.1020 ± 0.0048, significantly higher than that of the control (0.0500 ± 0.0017) (*t* = 33.2000, *p* < 0.001) (Figure 4c). This confirms that AQP L596_g7661 facilitates glycerol transport.

### 3.8. Response of AQPs to Osmotic Dehydration

**Figure 5:**
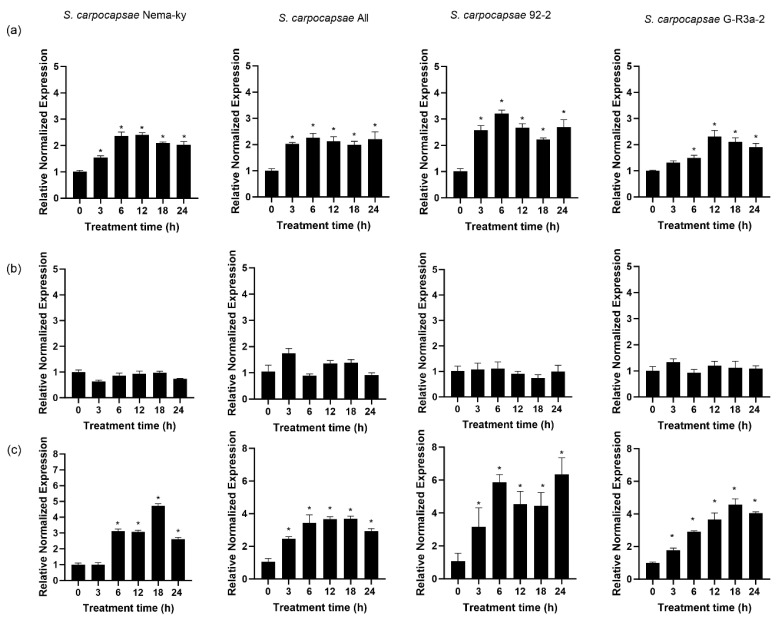
Relative expression levels of aquaporin genes in different *Steinernema carpocapsae* strains following osmotic treatment over time. (**a**), L596_g7661; (**b**), L596_g18121; (**c**), XLOC_007750. Asterisk (*) above the bars indicates significant difference between treatment time and the control (0 h) (Paired-sample *t*-test, *n* = 6, *p* < 0.05).

The expression levels of L596_g7661, L596_g18121 and XLOC_007750 were quantified in the four strains after 0 h to 24 h of osmotic dehydration (Figure 5). The same AQP gene exhibited similar expression patterns across different strains. The expression level of L596_g7661 increased significantly at all time points from 3 h to 24 h compared to the control (0 h) (*t* ≥ 4.46, *p* ≤ 0.047) (Figure 5a). The expression level of XLOC_007750 followed a similar pattern, being significantly upregulated compared to the control (*t* ≥ 4.364, *p* ≤ 0.049) (Figure 5c). In contrast, the expression level of L596_g18121 did not change significantly within the 24 h osmotic treatment period in any of the strains (*t* ≤ 4.173, *p* ≥ 0.053) (Figure 5b).

## 4. Discussion

Aquaporins are ubiquitous channel proteins belonging to the MIP superfamily, typically characterized by two NPA motifs and six transmembrane helices. They mediate the transport of water or/and other small solutes [23]. This study is the first to clone multiple different AQP genes from the same species of EPN. The fundamental characteristics of the cloned *S. carpocapsae* AQPs closely resemble those of *C. elegans* AQPs [13], supporting their predicted function in transporting water and small solutes like glycerol.

While nucleotide sequences showed some variations for the same AQP gene across different *S. carpocapsae* strains, the deduced amino acid sequence were highly conserved, all containing the critical NPA motifs—alterations in which can affect solute permeability [24]. All identified *S. carpocapsae* AQPs contain the conserved MIP domain with six transmembrane helices and intracellular N- and C-termini, a common architecture that facilitates the regulation of AQP activity [25]. The minimal amino acid variations observed among strains did not alter the conserved response patterns to osmotic stress, suggesting they do not significantly affect the core protein function investigated here. Whether these subtle variations lead to functional differences in other physiological contexts warrants further study.

Phylogenetic analysis using *C. elegans* AQPs as a reference placed L596_g7661 and L596_g18121 in a clade with *C. elegans* AQP1 and AQP7, and XLOC_007750 with *C. elegans* AQP8. As *C. elegans* AQP1, AQP7, and AQP8 are established aquaglyceroporins [13], it is inferred that the three *S. carpocapsae* AQPs also belong to this subfamily, functioning as water-glycerol channels. This inference was functionally validated for AQP L596_g7661, which demonstrated significant glycerol permeability in *Xenopus* oocytes, a hallmark of aquaglyceroporins. Glycerol plays an important role in rapidly balancing the osmotic pressure when IJs of *S. carpocapsae* were exposed in hypertonic solutions. IJs are induced to synthesize the protectant glycerol under osmotic dehydration, and glycerol permeates into the body of IJs during dehydration in glycerol solution. Part of the permeated glycerol acts as a protectant, similar to that synthesized by IJs from their energy reserve materials [26,27]. The significantly increased expression levels of aquaglyceroporins L596_g7661 and XLOC_007750 likely facilitate glycerol transport into the IJ body as a protectant. This reflects the essential presence of glycerol for IJs to survive and function properly even under moderate osmotic dehydration, especially when IJs are dehydrated in salt solutions. The low water permeability of L596_g7661 is consistent with other members of the aquaglyceroporin subfamily [12,28]. While this study provides the first functional characterization of an AQP (L596_g7661) in EPNs, future work should include functional assays for other AQPs (e.g., XLOC_007750) to further confirm the role of this subfamily in osmotic stress adaptation. Nonetheless, the conserved sequence and parallel expression response strongly suggest overlapping functions among these stress-responsive aquaglyceroporins.

The differential expression patterns of the three AQPs under osmotic stress—significant upregulation of L596_g7661 and XLOC_007750 versus the stability of L596_g18121—strongly suggest functional specialization within this gene family. Given that L596_g7661 and L596_g18121 are phylogenetically clustered yet display opposing transcriptional responses, we hypothesize that they may be expressed in distinct cell types with different roles in osmoregulation. For instance, the responsive AQPs might be localized to the hypodermis for rapid water/glycerol exchange with the environment, while the non-responsive L596_g18121 could serve a constitutive housekeeping role in internal tissues. This compartmentalization of function would allow the nematode to mount a targeted and efficient response to dehydration. The differential regulation suggests functional specialization, a phenomenon observed in other systems [29]. For example, in *C. elegans*, AQP1, AQP7, and AQP8 exhibit distinct tissue-specific expression patterns [13]. In the spiny dogfish (*Squalus acanthias*), AQP1 is expressed at very high levels in the rectal gland and at lower levels in other tissues [30]. AQPs displayed a tissue-specific expression pattern in different tissues of healthy spotted sea bass [31]. In buffalo and goat, some AQPs (AQP0, 2, 4, 5, 7, 9, 12) showed tissue-restricted expression, whereas others (AQP1, 3, 8, and 11) exhibited ubiquitous expression across several tissues [32]. Future studies should aim to determine the precise tissue localization of these AQPs in *S. carpocapsae*.

Building on the current findings, future work will focus on functionally characterizing the remaining AQPs, particularly the stress-responsive XLOC_007750, to define its specific solute transport profile. Furthermore, employing techniques such as RNA interference (RNAi) or gene editing to knock down target AQP genes in vivo would be essential to confirm their specific and indispensable roles in glycerol-mediated dehydration tolerance. Finally, investigating the tissue-specific localization and upstream regulatory mechanisms of these AQPs will provide a comprehensive understanding of their functions within the osmotic stress adaptation network of EPNs.

## 5. Conclusions

This study provides the first comparative functional analysis of AQP genes across different *S. carpocapsae* strains. The findings reveal the conserved stress-responsive expression patterns of specific AQPs and provides direct functional evidence that AQP L596_g7661 acts as a glycerol transporter, thereby offering crucial molecular insight into the osmotic stress adaptation mechanism in *S. carpocapsae*. This work ultimately paves new avenues for enhancing the application efficacy of EPNs in ecological pest control.

## Figures and Tables

**Table 1:** Primers used for gene amplification and quantitative PCR.

Primer Name	Primer Sequence (5′-3′)	Product Length (bp)	Usage
Sca7661-F1	ATGGAAGTGCTCCGGCG	864	PCR
Sca7661-R1	CTACTGTTCAAACTGCTTAAGAGGCAA		
Sca18121-F2	ATGCAGCTGCATCCCGACAAC	906	PCR
Sca18121-R2	TCACTCGGAAGAATCTTTGGCGAG		
Sca007750-F4	ACGGGTTAAAGTGTGGATGG	960	PCR
Sca007750-R4	ATGAATAGTCTCCGCGAGAC		
7661-HR-F	AGATCAATTCCCCGGGGATCCATGGAAGTGCTCCGGCGCAAG	894	PCR
7661-HR-R	CACCATGGGTACCTGTTCAAACTGCTTAAGAG		
7GFP-HR-F	AACAGGTACCCATGGTGAGCAAGGGCGAGGAG	751	PCR
7GFP-HR-R	CCAGATCAAGCTTGCTCTAGATTACTTGTACAGCTCGTCCATG		
Sca-actin2-F	TGTGACGAAGAAGTTGCCGC	81	RT-qPCR
Sca-actin2-R	AGCGTCATCTCCGGCAAAA		
aqp7661-qf1	TTACTACGACGCGATCCAGC	89	RT-qPCR
aqp7661-qr1	AGTCTCCAGGGTAGGAGCAG		
aqp18121-qf3	GGGTCAATTTCGGATGGGGA	149	RT-qPCR
aqp18121-qr3	AGCGATGCAGTAAACGACGA		
aqp7750-qf2	AACTCTACCTCAGCCCACCT	110	RT-qPCR
aqp7750-qr2	AAGGCAGTAAGGGAGATGGC		

**Table 2:** Physicochemical properties of aquaporins from different *Steinernema carpocapsae* strains.

Gene Name	Strain	Molecular Weight (kDa)	Instability Index	Isoelectric Point	Average Hydrophilicity
L596_g7661	Nema-ky	32.08	27.41	7.12	0.575
	All	32.05	26.98	7.12	0.578
	92-2	32.05	27.15	7.73	0.578
	G-R3a-2	32.08	27.41	7.12	0.575
L596_g18121	Nema-ky	33.17	22.15	6.37	0.463
	All	33.17	22.15	6.37	0.463
	92-2	33.11	21.67	6.70	0.465
	G-R3a-2	33.18	22.56	6.37	0.450
XLOC_007750	Nema-ky	33.19	21.58	7.05	0.445
	All	33.19	21.58	7.05	0.445
	92-2	33.21	22.55	7.05	0.403
	G-R3a-2	33.19	21.58	7.05	0.445

## Data Availability

The nucleotide sequences generated in this study are available in the NCBI database. The other original contributions presented in this study are included in the article/Supplementary Material. Further inquiries may be directed to the corresponding author.

## Supplementary Materials

The following supporting information can be downloaded at: https://www.mdpi.com/article/10.3390/biology15010078/s1, Figure S1: Conserved domains of aquaporins in different *Steinernema carpocapsae* strains; Figure S2: Predicted transmembrane domains of aquaporins in different *Steinernema carpocapsae* strains.
