# Supplementary figures and images for: Comparative Functional Analysis Reveals Conserved Roles of Aquaporins Under Osmotic Dehydration in Steinernema carpocapsae Strains
Source: Biology (Basel). 2025 Dec 31;15(1):78. doi: 10.3390/biology15010078 (PMC12784680; doi:10.3390/biology15010078)

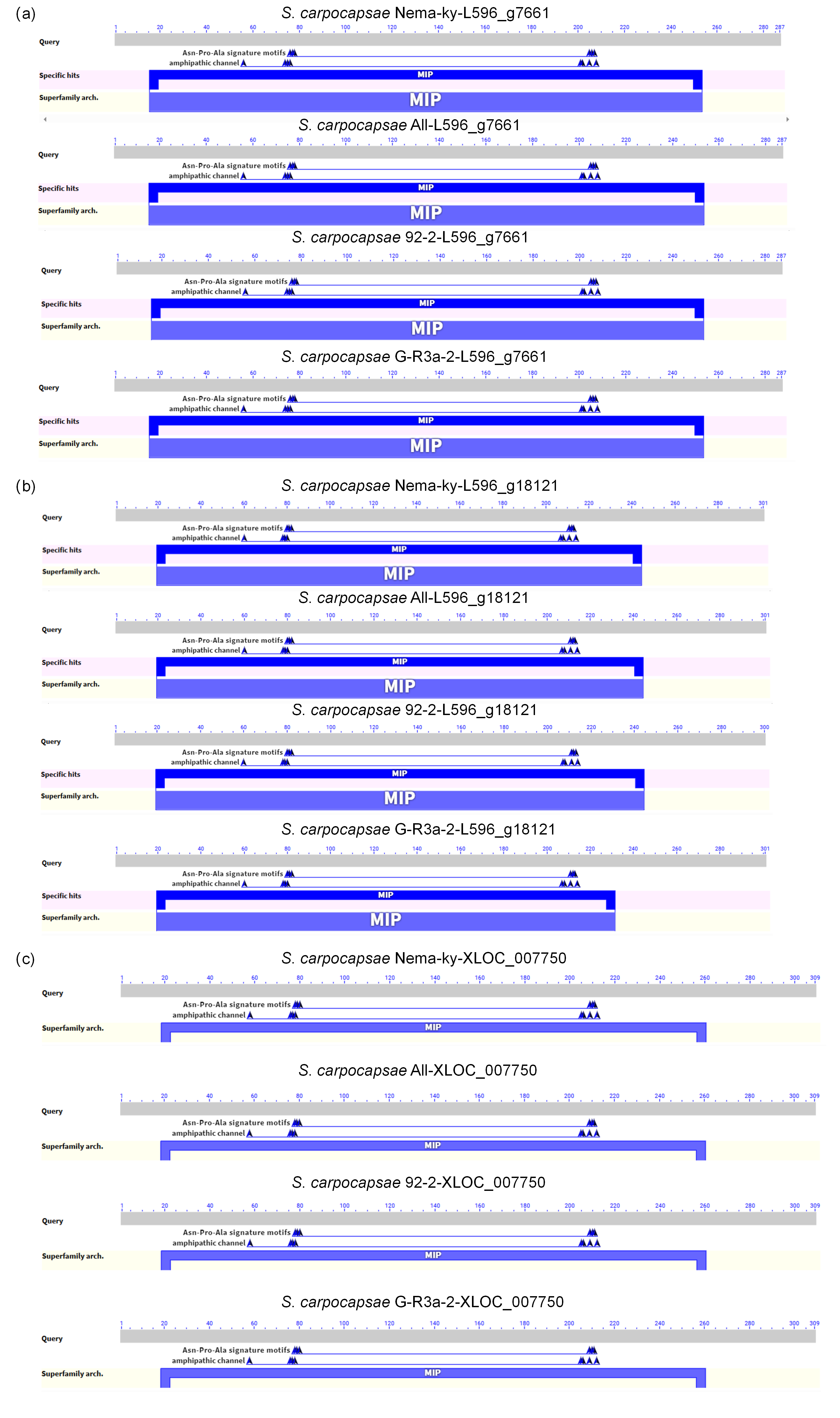

Supplement: Supplementary file 1 [file biology-15-00078-s001.zip › Figure S1 Conserved domains of aquaporins in different Steinernema carpocapsae strains.png]

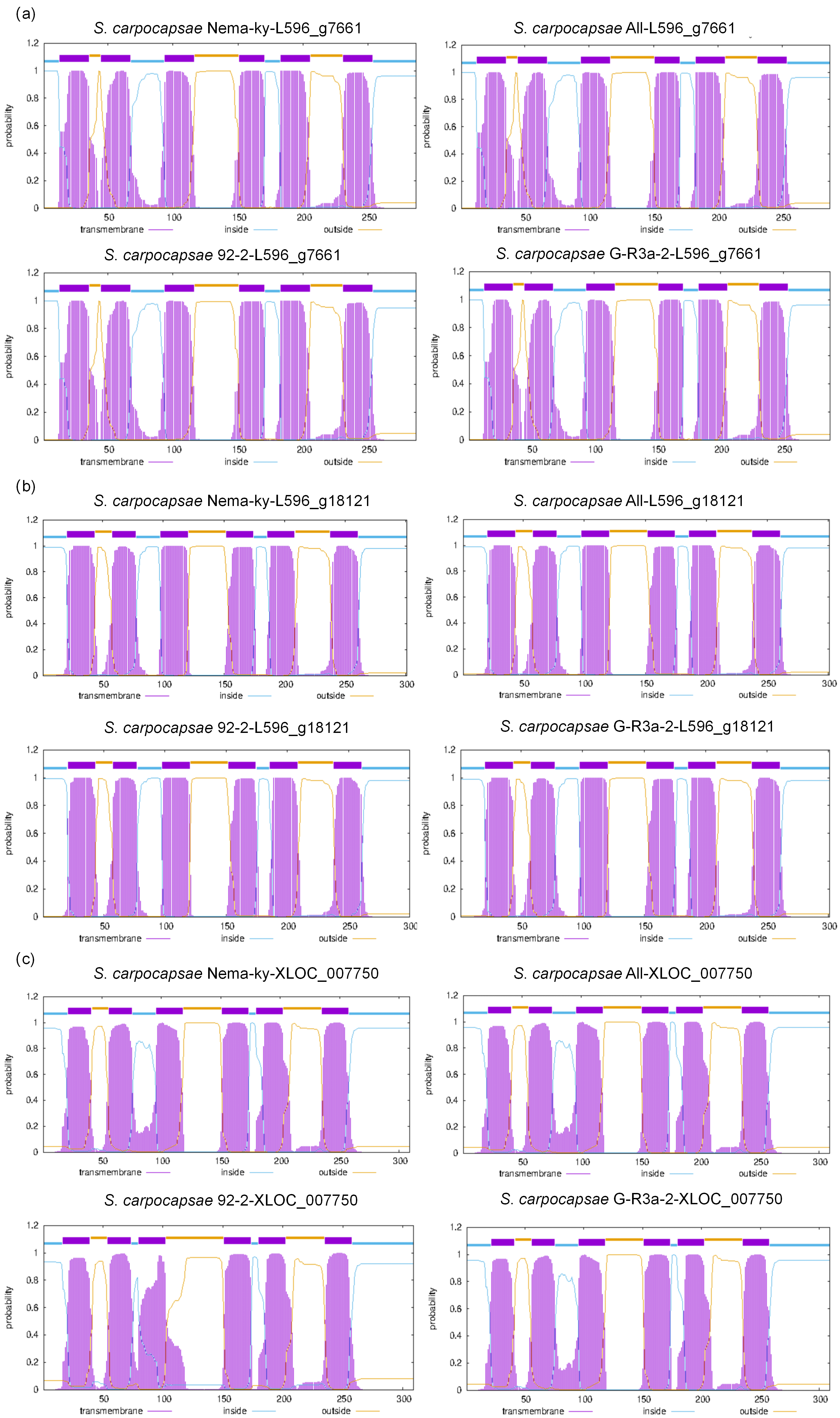

Supplement: Supplementary file 1 [file biology-15-00078-s001.zip › Figure S2 Predicted transmembrane domains of aquaporins in different Steinernema carpocapsae strains.png]
